# The Hospital Nursing Supplement

**Published:** 1893-03-11

**Authors:** 


"The Hospital" Nursing Mirror.

Being the Extra Nursing Supplement of "The Hospital" Newspaper.

[Contributions for this Supplement should be addressed to the Editor, The Hospital, 428, Strand, London, W.C., and should have the wor[illegible] "Nursing" plainly written in left-hand top corner of the envelope.]

### News from the Nursing World.

### PRINCESS HENRY OF BATTENBERG.

H.R.H. Princess Beatrice has consented to act as President of the Ryde District Nursing Association, and amongst the new annual subscribers the Queen heads the list with £10. The society was last year affiliated with the Q.V.J.I., of which Colonel Gildea is Secretary. The annual meeting, at which these facts were announced, was held recently in the Ryde Town Hall, and was largely attended.

### TRAINED NURSES' ANNUITY FUND.

The committee of the Trained Nurses' Annuity Fund, of which Princess Christian is President, records a serious falling off in annual subscriptions. This fact is accounted for by various unavoidable causes. However, there has been an increase in the donations during the year, as shown in the eighteenth annual report. There have been no deaths amongst the annuitants, and several names have been added to the number of candidates. The funds of the society do not at present permit of its increasing the number of annuitants to whom the grant of fifteen pounds per annum can be made.

### TOO LATE!

We beg to remind Secretaries and others in whose hands these arrangements lie, that unless notices of meetings reach us in good time it is quite impossible for us to send reporters to all the institutional gatherings which are of interest to our readers. Those who are responsible for sending out the cards will do well to give as long a warning as possible, otherwise we are reluctantly obliged to pass over many meetings and festivities.

### SENSE WITHOUT SYRUP.

An experienced nurse writes this week in another column on the wholesale administration of so-called soothing syrups. The *British Medical Journal* gives a report of a lecture on "Cruel Advertisements," in which the Rev. Horace Waller strongly denounced quack remedies. Surely poor little babies have enough to bear from the many "ills which flesh is heir to," without being drenched with unreliable medicines! Those fully-trained nurses who develop into good health lecturers find an enormous sphere of usefulness. They have so many opportunities of offering seasonable counsel to the inexperienced girls and untaught mothers, in whose hands lie infants' lives, and who will often listen with patient intelligence to "the lady from London," who comes "a purpose to talk to we." Much tact is needed to sow a crop of common sense which will eventually bring forth a harvest of wisdom. Reasonable attention to the laws of health will some day drive out the pernicious habit of indiscriminate drugging of both babies and their elders. District nurses have it in their power to largely influence the mothers who "see no harm" in giving patent medicines on their own responsibility.

### CONVALESCENT FROM FEVER.

All people know the difficulties attendant on the convalescent stage of scarlet fever. When change of air is urgently needed for patients, it is well-nigh impossible to secure it without some risk of infecting other persons. Yet convalescent homes for fever cases are, indeed, few and far between. Leeds has recently provided itself with an admirably-situated establishment, which has accommodation for 20 patients, convalescent from scarlet fever. The house stands in 24 acres of grounds, and has a lovely view over open country. It will be well when every large town starts a similar institution.

### A NEW HOSPITAL.

A little hospital, opened this week at Bradford, has schemes for unlimited good work before it, and everything has been arranged with a view to carrying out the nursing in a worthy spirit. St. Catherine's Hospital for Cancer has a beautiful day-room for patients who are able to leave their beds, and the arrangements in every department do credit to the kindly consideration of the ladies by whom the establishment has been planned. Under the management of Miss Hadley-Scott, and supported by a committee who intend their hospital to be a perfect one, there is little fear of disappointment being experienced in any one particular.

### NURSING AND NURSED.

The Birmingham and Midland Counties Training Institute for Nurses held its annual meeting last month. The influenza epidemic at the beginning of last year had attacked some of the nurses, and had not only incapacitated them from work, but had also made it necessary for others to be retained in the home to attend on the sufferers. Notwithstanding this, there remains a record of excellent work done in the town and neighbourhood by the eighty-two trained nurses of whom the staff is composed. Ten probationers have completed their training at the Birmingham General Hospital, Wolverhampton General Hospital, and the Worcester Infirmary. The Pension Fund now amounts to close on £3,000, and the institute also provides six nurses for the District Nursing Society.

### OUT-DOOR RELIEF.

The Cambridge Nurses Home, supported entirely by voluntary contributions, ensures to the sick poor free and efficient nursing in their own homes. Including the Lady Superintendent, the staff now consists of seven nurses, who are doing excellent work. At the annual meeting last month various acceptable gifts were notified, and a most satisfactory report presented. The Guardians, with the sanction of the Local Government Board, have increased their subscription to £50, and are able to secure the services of a nurse at any time on application being made to the Lady Superintendent by the relieving officer. This arrangement has apparently worked well, and for eight months last year a nurse was thus supplied. Evidently the Cambridge Guardians set a proper value on skilled nursing.

### CHIPPING NURSES' HOME.

Wotton-under-Edge has four qualified nurses and two probationers, whose services are of great benefit to the town and neighbourhood. They are shortly to be transferred to new premises, a convenient Home at Chipping being now furnished for them. It is under discussion to add two beds for patients to the new establishment.

### AT HOME BY THE SEA.

A house standing in three acres of garden ground within five minutes' walk of the sea is but a simple description of a charming home for invalid ladies and gentlemen. Miss Taylor, Wick Lodge, Brighton, is fortunate in her house and happy in the support of seventeen eminent physicians and surgeons who allow their names to appear on her prospectus. Anyone of them would be looked upon as guarantee for the health-giving properties of a spot thus recommended. Miss Taylor not only invites patients to take their own nurses, but she offers accommodation for carriages, and also provides such comforts as render a sojourn in her establishment a continuance of luxurious home life.

### DR. JAMES ANDERSON.

Last week The Hospital announced the death of Dr. James Anderson, and the *British Medical Journal* and the *Lancet* have recorded the appointments and the honours, which rewarded perseverance and continuous hard work such as but few men accomplish in far longer lives. Dr. Anderson's labours were not confined to his profession, for to his own affairs he constantly added those of others, taking their burdens and their troubles upon himself, and never failing to lighten, when he could not remove, the load. One cannot but fear that, unconsciously, many attached friends let their own trials press heavily on the brave spirit which was ever ready to share its strength with the weak, the sick, and the poor. Always occupied, he was never too busy to spare time and sympathy for the humblest claimant. On the evening of March 2nd a large number of doctors and other friends assembled in Wimpole Street to pay their last respects to his memory. The oak coffin was hidden by beautiful wreaths, and those sent from the London Hospital, which was always so dear to his heart, were placed on it first. The Medical Staff, the Students, the Committee, the Sisters, the Nurses, and the Porters each sent a beautiful tribute, and there were a number of other wreaths from various friends. At King's Cross, more doctors and students waited in reverent silence, with uncovered heads, for the coming of one who, "being dead, yet speaketh." Dr. Anderson's sisters and brother were closely followed to the train by many members of the visiting staff, and several London Hospital sisters and nurses. When the carriages glided slowly out of the station on the long journey to Aberdeen, the silence amongst the spectators showed the truth of the words, "Light griefs are loquacious; deep sorrow hath no tongue." Dr. Anderson is laid in the same grave with his mother, who died a year ago. The cemetery is a short distance outside Aberdeen; a lovely spot, overlooking the sea, where a vast concourse of old University and other friends were present at the funeral on Friday morning.

### HULL ROYAL INFIRMARY.

Several very welcome improvements have taken place at the Hull Royal Infirmary of late. Miss Cox, the Lady Superintendent, rejoices to report the disappearance of the old flock beds, always such trials to nurses and patients, and now superseded in the infirmary by excellent hair mattresses. The library contains upwards of a hundred books, and is attracting the interest and support of many friends, besides being an immense benefit to the nurses, for whom Miss Cox has taken such pains to develop it. It is always pleasant to hear of progress of this kind.

### REASONABLE REGULATIONS.

The *Madras Times* records that the designation of "Acting Superintendent" has been altered to "Deputy Superintendent" in the Indian Nursing Service. Another change has been made by the Government of India to the effect that when the plea of ill-health is advanced by a nurse in support of an application for leave to retire, the opinion of a medical board must be obtained in support thereof. This seems a perfectly fair arrangement, and so does the one which requires that a lady resigning her appointment within the specified time (five years) for any cause, save ill-health, shall refund her passage-money. No conscientious workers will be deterred from joining the Indian Nursing Service by such obviously just stipulations on the part of the Government.

### NURSES FOR INDIA.

Everybody has something to say about nursing in India, and just now the proposed up-country Association for Nursing Europeans is very much to the fore. Firstly, funds are wanted. The patients who will benefit by the society are chiefly persons with limited incomes and many claims on them. Travelling and other expenses add considerably to the cost of a nurse in India, and without doubt it would be an advantage to have outlying stations better provided with help. Sickness in India is generally sudden and serious. The second point is the selection of suitable nurses for this very responsible work, and it is one which deserves to be thoroughly considered. The *Bristol Times* suggests that the nurses wanted for this field of labour are "those who are anxious to combine real philanthropy with their means of livelihood, and not women who are mainly concerned in getting money or a husband. The market is already supplied with that class. But for skilled nurses who are devoted to their noble calling there is great demand in India."

### SHORT ITEMS.

Nurse Tait has been appointed Q.V.J.I. nurse for Buckie.—Leeds employs ten trained district nurses for the sick poor, and branch homes are soon to be opened in Holbeck and Hunslet.—During 1892 the East London Nursing Society ministered to nearly 4,000 poor people; the society has four matrons and twenty-nine nurses.—On the completion of a course of lectures on "Home Nursing" to the Scotch Girls' Friendly Society, Dr. D. Carmichael was presented with a handsome easy chair from the members of the class, by whom his instruction has been much appreciated.

### THE DISABLED NURSE.

We have this week received for our invalid sister: Matron, 2s. 6d.; Nurse Wheeler, 5s., making a total of £10 19s., of which £2 7s. 6d. has been handed to the invalid.

### "THE HOSPITAL" ENDOWED BED.

We beg to remind our readers that subscriptions for the Nurse's Bed are now due. All contributions sent to the Editor, The Lodge Porchester Square, will appear in The Hospital. Miss Yorke has forwarded £1 5s., making total £13 1s.

### Management of Consumption.

### V.—SWEATING AND DIARRHŒA.

*Sweating.*—This symptom is by no means constant in consumption. Some cases may be watched from beginning to end without there ever being observed sweating to any considerable extent, while others sweat profusely from the first. Most consumptives, on being interrogated on the subject, will admit to sweating, but on closer inquiry it will be found that frequently this is only slight, and differs considerably from the genuine sweating of consumption. When a consumptive sweats there can be no mistaking it, both from its character and degree of intensity. A true sweat of consumption is somewhat as follows, the patient in the evening is hot and feverish, the brow and lip are bedewed with slight drops of moisture; the cheeks are flushed, especially over the cheek bones; the patient, after perhaps a violent fit of coughing, falls asleep. If one now goes gently to the bedside and watches the forehead and lips of the sleeper, the drops of sweat will be seen from time to time to increase in number, until they flow away to the sides of the head, and in time soak the hair of the patient and the pillow; in a few hours the head (and the pillow on which it is lying look just as if they had been drenched with water. If the bedclothes be turned back, and the hand placed beneath the nightdress and the chest, the same things can be made out; the chest will be quite wet, and in time the nightdress is quite soaked, and if the trouble be taken, the water can sometimes be positively wrung from the garment. In this way the whole body, from the crown of the head to the soles of the feet, is bathed in a profuse sweat. The patient then wakes up, and finds himself in this truly deplorable state. His usual term of expression when describing a night sweat of this kind exactly hits the mark, for he says when he wakes, one would think he had been lying in a pond all night. This great drain upon the patient's system shows its effects, for instead of being refreshed, he is languid and weak after his night's repose.

With the sweating comes a fall of temperature. Frequently the bed, instead of being hot or warm, feels damp and cold, and the sweats are then, not inaptly, called cold sweats. At times the sweating is so easily produced that the moment the patient takes a nap it comes on at once, and often even a violent fit of coughing is enough to start it. This, then, is the true night sweating of consumption; but there are other kinds of sweating among consumptives which are of trifling moment. As is well known, consumptives are in the habit of clothing themselves with thick woollen clothing to guard against the dreaded chill; they sleep in these, and the consequence is they sweat, but this is a very different thing, and is never profuse; it is always hot, and it always ceases when the clothing is made lighter. Heated rooms, more blankets than are really necessary, and feather beds are conducive to this spurious kind. In treating sweating, the first thing to do is to determine if having the patient lightly clothed when in bed will remove it. The nurse must see that no merino vests are worn under the nightdress, and she must regulate the amount of bed clothing, a good thick blanket and a sheet being all that is required in summer time; in winter another blanket and quilt may be given. The nightdress should be of linen, and, if possible, the sheets should be made of twill. In hospitals patients should have an extra nightdress, and as soon as one is soaked the dry one can be put on; in this way much danger from catching cold is avoided. There should, indeed, be a double supply of pillow-cases and sheets, in addition to the sleeping gowns, for it makes all the difference in the world to the comfort of the patient to have a change of linen when necessary. The nurse should frequently test the condition of her patient's skin by placing the hand on the chest She can thus know how to regulate the amount of bed clothing without disturbing the patient. There are a multitude of ways of treating night sweating; it may often be controlled by sponging the patient over before going to bed with tepid water, and vinegar and water, and with a fairly strong infusion of cold tea. The tannin in this latter fluid has an astringent effect upon the skin. Sometimes the moment the patient lies down the sweating comes on, and in some cases, where it has persisted in spite of all treatment, making the patient comfortable in a semi-recumbent position in an easy chair, has at once controlled it. Of drugs, the most used is belladonna, and its active principle, atropine. This drug is generally effective, but has one drawback, if taken for a considerable length of time it produces dryness at the back of the throat and disorders of vision, so that it is well to be on the look out for these two symptoms. Oxide of zinc, gallic acid, aromat.c sulphuric acid, and picrotoxin are all used with good effect for this unpleasant symptom, and it is the rule for night sweating to improve under such tr atment.

*Diarrhœa* often complicates consumption, and when it does is due to irritation caused by ulceration of the bowels. This ulceration is due to the same cause as consumption, viz., the bacillus tuberculosis. The intestine becomes infected, either by means of swallowed expectoration, or else by means of the blood. The importance of not permitting patients to swallow their expectoration has already been insisted upon. If there is reason to believe that the intestines are affected with tubercular ulceration, all the evacuations should be received into a strong solution of perchloride of mercury, of strength 1 in 500, and then thrown away; and all linen which is soiled should be treated in a similar manner before being sent to the wash. The diet of such patients should be much the same as has been already advised for a patient with severe vomiting. Unfortunately this complication, when once fully established, is amenable to hardly any treatment. The case goes down h ll, and in no long time ends fatally. The part of the nurse here is evidently to attend to directions as to antiseptics and feeding.

### The International Congress of Charities, Correction, and Philanthropy.

We have much pleasure in publishing the following invitation of Miss Hampton, Chairman of the Nursing Section of the Chicago Congress, which has been issued to lady superintendents and matrons of British hospitals and nursing institutions. We learn that, evidently by some inadvertence, the lady superintendents and matrons of a few institutions have not yet received a copy. We shall be obliged if anyone so situated will send us word at once, and meanwhile kindly understand that their letter has either miscarried in the post, or been accidentally delayed. We venture to hope that all who feel moved to write on some practical question will send papers to Miss Hampton, and that the heads of the leading and most representative nurse training schools in Scotland and Ireland, as well as in England and the metropolis, will not only send papers, but encourage representative members of their nursing staffs to do the same.

Rutherford B. Hayes, *President;* Frederick H. Wines, *First Vice-president;* Robert Treat Paine, *Second Vice-president;* Alexander Johnson, *General Secretary. Committee of Organisation:* Frederick H. Wines, John G. Shortall, Mrs. T. M. Flower, Nathaniel S. Rosenau, *Secretary.*

Section on Hospital Care of the Sick.—Training of Nurses, Dispensary Work, and First Aid to the Injured.

John S. Billings, M.D., U.S.A., *Chairman*, Washington, D.C.; Henry M. Hurd, M.D., *Secretary*, John Hopkins Hospital, Baltimore, Md.

Baltimore, Md. U.S.A., January 31st, 1893.

Miss ………, *Matron.*

Dear Madam,—Section 3 of the International Congress of Charities, Correction, and Philanthropy treating on the

"Hospital Care of the Sick, the Training of Nurses, Dispensary Work, and First Aid to the Injured," will be held in the City of Chicago, June 12th to 18th, 1893. The sub-section on the Training of Nurses will take part in the General Sessions and in two sectional meetings, and in addition will hold three separate meetings, June 13th, 14th, and 17th, for the purpose of hearing papers and discussions on nursing subjects.

As this is our first meeting together as a body of professional women, it is desirable that the women who have large experience and knowledge of nursing, and who understand the problems and needs of nurses' work, and are interested in their progress and development, should by the influence of their presence and by contributions or discussions make the Congress not only a present value to nurses, but the beginning of future unity and closer intercourse and benefit. We shall be glad to have you take part in the discussion on "Training Schools in Great Britain," or if you prefer to prepare a paper on any subject we shall be glad to receive it. Hoping for a favourable reply by an early date, believe me, yours faithfully,

(Signed) Isabel A. Hampton,
*Chairman of the Sub-section.*

### Asylum Nursing.

By a Member of the Staff of the Graham's Town Asylum, South Africa.

The subject of asylum nursing, and of raising the tone of asylum attendants, has been so much under discussion lately that I have felt a desire, as an asylum nurse of nearly five years' experience, to say a few words also upon the matter. I have in my own mind a very definite idea of what a good mental nurse should be, and I know full well where and why I have myself failed in coming up to this standard; I therefore think I may venture to offer a few hints to those who are starting on their career as asylum nurses.

Education, in the form of lectures and examinations, may be useful in its way, chiefly because it teaches us that insanity is really a bodily disease, dependent on bodily conditions; but the most accurate knowledge of the structure of the brain will not give a mental nurse the tact and patience which are her necessary equipments, and if she be lacking in these no amount of technical training will supply their place.

I have often thought that the first thing a nurse has to learn (and sometimes the last thing she succeeds in learning) is to realize that the patients under her care are really insane. I am not going to offer any fresh definition of the word insanity, which has been so many times explained already, but I can best make my meaning clear, by saying that an insane person is one who has lost his self-control—not entirely, because many insane people exercise a great deal of self-control; but still, in one or more important respects, his words and actions are no longer controlled by his reason; and this is his disease, which he can no more help than he could help being consumptive or having a weak heart.

The old maxim, "Put yourself in his place," will apply here as well as anywhere. We must try, by careful observation, to discover the patient's state of mind, and then to imagine ourselves in the same position. For instance, a patient who is constantly trying to escape, is a very wearing and anxious charge; but suppose that you and I found ourselves suddenly shut up in a place which was not our home, among a number of people who were perfect strangers to us, deprived of our liberty, and not knowing the reason why, should we not also make every effort to escape?

Thus, the mental nurse requires to be sympathetic fully as much as the sick nurse; we must enter into th feelings of our patients if we are to do them any real good.

Lunatics are in many respects like children. They can be spoilt by indulgence, and they can be much improved, even where there is no hope of recovery, by wise discipline. But this discipline, which in every asylum depends in great measure upon the nurses, should never be allowed to include the idea of punishment. It is quite necessary to have the mastery over your patients, and to make them feel it; but the nurse who pulls a patient's hair because her own has been pulled, or who gives a patient a good shaking because she herself has received a blow or a kick, is merely exhibiting her own want of self-control, and does away with the good effects of her previous victory.

I once overheard a conversation between two nurses which will exactly illustrate my meaning. The one observed that a patient had struck her in the face, whereupon the other asked, "Did you strike her back again?" "No," replied the first nurse, with an expression of surprise; and when the other said "Why not?" she rejoined quietly, "Because she is a lunatic, and I am not."

Herein lies the gist of the whole matter. Directly a nurse allows herself to repay a patient in her own coin, she lowers herself to the level of the patent; and the patient is the first to find it out.

But many nurses who are quite able to control their hands find it much more difficult to control their tongues; and here, indeed, we all have a very trying task. I do not refer to those nurses who seem to think that because their patients are insane they may speak to them as if they were dogs. These belong, let us hope, to a fast diminishing class. But while few nurses are annoyed by the violent and incoherent abuse of a raving lunatic, not many can take with invariable patience the cool and deliberate insult with which many insane persons continually wear the tempers of those in charge of them. It is just because their outrageous rudeness is so coherent and sensible that it is so irritating; and yet, if we consider it rightly, it is the very proof of their insanity which we require in order to know how to deal with them. If, for instance, the patient is a lady, we must know that in her right mind she would never have dreamed of using such language to anyone; therefore, her rudeness is, after all, only a sign of her disease, and to reply to it, as too many nurses do, wich equal rudeness, is only a proof of ignorance and inexperience. To speak sharply is often necessary; to speak rudely, never; and a good nurse can always be sharp without being rude, just as she can hold a patient firmly without being rough. In a word, she must be distinguished for that very quality in which the patient is lacking—the power of self-control.

I have already said that, in order to be in touch with her patients, the nurse must carefully study the character and peculiarities of each one placed under her charge. It is true that to a casual observer the majority of lunatics are very uninteresting, but to those who live with them, every patient, however imbecile, will present some features of interest to repay observation; and it is only by the continual practice of this observation that we learn to guide ourselves in such important matters as the following: What is most liable to irritate a patient; what is most likely to upset and excite him, and so to retard his recovery; when it is best to take notice of his delusions and tempers, and when it is best to pass them quietly by unnoticed; when it is of any use to reason with him, and when the only wise course is to try and divert his mind from the insane ideas which govern it.

It is only by such study of our patients that we gain really useful experience; without it a nurse may live twenty years in asylums, and not gain any experience worth the name.

There is a very large class of insane persons whose most aggravating characteristic is the way in which they "put on." Every nurse knows the hysterical lady patient, who lies in bed and feigns illness simply to attract the notice of he doctor and the matron (especially the former), and to monopolise the attention of the nurses, and who, directly she finds that no fuss is made about her, or hears that an entertainment is going forward, recovers with surprising rapidity, and in an hour or two is perfectly well.

Now these are the patients of whom the most experienced nurses invariably say, that "they can help themselves." In one sense they are right, but in another they are quite wrong. The hysterical patient cannot help herself, because she is really insane, and her ridiculous and morbid affectation is the chief symptom of her insanity; but she can be made to help herself, and the way to do it is to treat her as if she could help herself. If she is noticed and made much of, she will of course go on from bad to worse; but if she is argued with and scolded, the effect will be no better; she does not in the least mind exciting anger and opposition, so long as some kind of notice is taken of her, some kind of fuss made about her, she is satisfied, and any roughness on the part of the nurse will at once furnish her with a fine opportunity for making a martyr of herself, and preparing a long list of complaints and grievances to pour into the ear of the doctor or the matron.

A judicious nurse will simply leave the patient alone, showing that she does not consider her more important or more deserving of attention than the other patients. She will thus take away from her any motive for "showing off," by teaching her that she has nothing to gain by it, in other words, she will "make her help herself."

And here I would say again, with these most irritating cases, "Silence is golden." If you cannot trust yourself to answer your patients civilly, answer them not at all; for if you once lower yourself to wrangle with them, your influence over them is gone.

Look at it as you will, the care of lunatics is a thankless task at best. Gratitude and affection are rarely found among them, and those who undertake the charge must look for much work and little reward. I often call to mind certain words of that unequalled student of human nature, Thomas à Kempis—words which every asylum attendant may lay to heart, for to no one could they apply with greater force: "To be able to live peaceably with hard and perverse persons, or with the disorderly, or with such as go contrary to us, is a great grace, and a most commendable and manly thing."

### Everybody's Opinion.

[*Correspondence on all subjects is invited, but we cannot in any way be responsible for the opinions expressed by our correspondents. No communications can be entertained if the name and address of the correspondent is not given, or unless one side of the paper only be written on.*]

### THE YOUNG NURSE ON HERSELF.

"A Young Nurse" writes: I have been reading with great interest the discussion on young nurses, as I went first into the work at seventeen, and my sister at sixteen and a-half. Certainly, we were taken as a favour, but still I think "age has nothing to do with it, it is ability;" these are the words of a shrewd Scotch doctor. At the age of nineteen I was made charge nurse of two wards, having under my care from 68 to 72 patients, and the doctor said I was quite competent. There are many people more fit for mothers and nurses at twenty than others are at twenty-five. Then again, reference has been made to helpless patients, and I think young, though not thoughtless, nurses are best for these. They have more life in them, and can make the patients forget themselves. My sister and I have been the life of the wards, and the patients expressed regret when we left them. It is indeed a pitiful sight to see helpless patients, especially young people. I had one such case, a youth of eighteen, with stiff joints, the result of bad nursing in rheumatic fever, who left us much relieved, and wrote to me. "Dear Nurse,—I should like you to be here to nurse me; I like you, and could not wish to be better nursed." There was another nurse three or four years older, whom he used to swear at and declare she handled him as though he had no feeling. Since I left, many have expressed a wish, with almost their last breath, that I had been there to nurse them. One old man said if he had a fortune he would leave it to me to repay me for my kindness. These are only a few instances out of many, but they show how I performed my duty. A paragraph from a testimonial received from the sister of a private case I nursed says, "I have much pleasure in stating that Nurse M—— performed her duties in a perfectly satisfactory manner, and my sister expressed herself as being greatly pleased by her uniform gentleness and consideration." I do not think war should be waged against young nurses who know their duties and perform them.

### AMERICAN EXPERIENCES.

"Nurse S." writes: "An Interested Nurse" wished for some information about America, and although "A. E. I." has already kindly answered, I think, perhaps, my experiences may be also of use. Knowing no one in America, but wishing very much to go to that country, I was advised to write to the Superintendent of the Young Women's Christian Association Institute at Quebec, the town I wished to reach, and to ask her advice as to whether a nurse was wanted in that city, stating my qualifications, &c. I received a kind and encouraging reply, requesting me to state the date of sailing of my steamer, so that I should be met on arrival. Those who know what it is to reach a strange city in a far away land, will appreciate the advantage of being welcomed by a kindly woman, anxious to render every assistance.

I brought out no introductions with me, and generally found an American doctor did not care much for them; and often when I was going to show my certificate I was stopped by his saying, "Don't trouble, I will give you one case to judge you by. I can then tell if you are a good nurse, if not, ten certificates would do you no good." I quite agree with "A. E. I." as to the kindness of the doctors. I had a nurse friend who was ill, and was not only tended with unceasing care, but during convalescence was taken to their own house by the doctor and his wife for a fortnight. Afterwards they lent her money to go for further change of air.

Boarding and lodging at the Y.W.C.A. Institutes and the Girls' Friendly Lodges cost much less than anywhere else. At Springfield, Mass. (about two hours from Boston), at the institute there, I was only charged three dollars (12s.) for board. A room shared with another was a dollar more, or a room to oneself, two dollars. The institutes are generally situated in the best streets and the most central part of the town; the reading, dining, and sitting-rooms are large and comfortable. There is always a piano, and the meals are good and plentiful. Another advantage of staying at these institutes is that nearly all of them have registry offices attached. I was not a day in Springfield when a lady came for a nurse, and I was sent. The Superintendent, who knows most of the doctors, will go round and introduce you to them.

I should advise nurses not to go to big towns, such as Boston or New York, already they are getting overstocked with nurses, and do not offer so good a field as the smaller places, or those further West.

### NURSES IN CANADA.

"Monthly Nurse" says: I am hoping to go to Ontario, Canada, in a few months to join my husband, and will be glad of some advice as to whether English nurses are appreciated in Canada, will there be any chance of my getting work there, and what fees are paid? Should I get on better if I had the L.O.S. diploma? Any information will be gratefully received.

### INFECTION IN HOLLAND.

"A Guy's Nomad" says: In "Scraps and Gleanings" of your last number attention is called to "An excellent custom the Dutch have," of tying a piece of white rag round the bell handle of any house in which an infectious illness occurs. I notice that this "excellent custom" has been replaced by another, more modern. A printed paper notifying the disease by which the house is infected is affixed to it, by order of the magistrate on receiving from the doctor the necessary notification, and it is only by his order that this may be removed.

### WELCOME GIFTS.

Sister Katherine writes: The Matron of Westminster Hospital has just sent us a number of old jackets. Might I ask you to mention this in your paper, as we should be so very grateful if other hospitals would follow this example. Such things are invaluable for our district work, and are quite good enough for our poor patients when considered too bad for ward use. All contribution sent to St. Mary's Home, Plaistow, will be gratefully acknowledged.

### CO-OPERATION FOR NURSES.

Nurse Olive writes: I was deeply interested in the account of the annual meeting of the Nurses' Co-operation in The Hospital. Why cannot the nurses of Birmingham have a similar association? There are many in this large city nursing on their own account, and it would, I feel sure, be a great advantage to the public and the doctors, as well as to the nurses themselves, if we could co-operate. Then the demand and supply would be in touch. Many nurses live in lodgings, and would greatly benefit by a central home between their cases. Surely Birmingham might be looked upon as a very good centre for such an association. Will you kindly make this suggestion public by means of your valuable paper?

### MORE CO-OPERATION.

"An Old Nurse" writes: The Matron of Beresford House, Beaumont Street, in addition to her nursing home, is starting a club for trained nurses working on their own account on the co-operative system—that is to say, they will take their own fees. They can have accommodation between their cases and board at a fixed rate by the week or single meal. Nurses wishing to arrange for a permanent home can secure this by special arrangement.

### CARBOLISED SHEET.

Nurse Mary says: No one seems to offer any help by suggestions such as The Hospital invited. Would not the idea of a "Drip-pot" come in? Some syphon arrangement. An overhanging violet leaf can keep a large circle of moisture on a table-cloth where it is not wanted. If a tiny vase of flowers unexpectedly acts thus, surely some intelligent nurse will see her way to carry out and describe an equally simple and successful arrangement?

"A. E. I." writes: After the sheet has once been saturated, one corner should be immersed in a pail containing the same solution of carbolic, and in this way it can be kept constantly damp without either mop or syringe.

Nurse Carmichael says: In reference to keeping disinfecting sheet over door permanently damp, I have found help from a spray, and also a small watering can is useful for the purpose.

Sister Lucie writes: Obtain some white art muslin at 8½d. per yard, or some old art muslin curtains from which all colour has been washed. Join them to make 8 in. beyond the actual width of the door frame, and 4 in. longer. Sew a loop of tape at each end and one in the centre of the top of sheet. Fix three large nails in the top of the framework above the door at distances to correspond with the loops on the sheet. Fold the muslin and place in an old towel, and proceed as for an ordinary fomentation, being careful to only pour out as much lotion as will be required each time. It is advisable to have two sheets, one in readiness to replace the other as soon as it becomes dry. The muslin answers quite as well as the usual heavy sheet, and is far less bulky to wring, though requiring to be more frequently attended to. It can be quickly and quietly done, and it is preferable to have the sheet inside the door of the patient's room. A nurse's hands constantly in 1-20 carbolic become very rough and unpleasant, and a patient will appreciate the method I suggest, for every invalid likes the touch of a smooth soft hand.

### "THE HOSPITAL" ENDOWED BED.

"A Correspondent in the North" writes: I enclose my subscription for the Nurses' Bed. We find the distance quite too far away for the bed to be of any use for our nurses when ill. Last year we had to send several needing change of air somewhere more convenient.

### Royal Edinburgh Asylum, Morningside.

### Presentation to Matron after Twenty-five Years' Service.

On Thursday, March 2nd, a dance was held for the patients, and at an attendants' dance on the following evening Dr. Clonston, in the name of the staff, presented Mrs. Macdougall with an elegant silver tea service and cake stand. Dr. Clonston spoke kindly of Mrs. Macdougall, saying she never sunk the woman in the official, but, like a mother, had a place in her heart for every one of her large family. Dr. Clonston also said he was proud of the fact that the term of office of his present principal officials averaged ten years. He impressed upon his staff the great importance of their work.

### Notes and Queries.

### Queries.

(66) *Out-patients.*—Can you tell me of Throat and Ear hospital for a boy?—*Nurse E. A.*

(67) *Massage.*—Information wanted as to libraries.—*E. A.*

(68) *L.O.S.*—When is the next examination of this Society?—*Bedford.*

(69) *Stockingette.*—Will you kindly tell me where the specially woven continuous tube can be procured? It was described in an article on Spinal caries, pape 317, in The Hospital, of February 11th.—*Sister.*

(70) *New York.*—Can you recommend me an American nursing paper? I also want information about institutions in American towns.—*T. McL.*

(71) *Technical Terms.*—Will you recommend me a simple book for reference as to medical terms?—*Nurse May.*

(72) *Abroad.*—Can you tell me the name of another nursing institute besides the Hollond one?—*E. A. H.*

(73) *One Year's Training.*—I shall be glad to know of a good hospital in London where I can get one year's free training in preparation for district nursing.—*J. S. R.*

(74) *Would-be Probationer.*—Can you inform me whether a First-Aid Ambulance Certificate would help me in obtaining a situation as probationer.—*Kate.*

### Answers.

(66) *Out-patients (Nurse E. A.).*—You will get all information in Burdett's "Hospital Annual," 428, Strand.

(67) *Massage (E.A.).*—The Trained Nurses' Club has a library at 12, Buckingham-street, Strand. You can write to Hon. Secretary for particulars. Kimpton, Holborn, and Lewis, Gower Street, have medical libraries.

(68) *L.O.S. (Bedford).*—Apply for all the information you require to the Secretary, 54, Berners Street.

(69) *Stockingette (Sister).*—From Gardner and Son, Forest Road, Edinburgh, 1s. to 1s. 3d. per yard.

(70) *New York (T.McL.).*—"The Trained Nurse," published at 202, Broadway, New York, 20 cents monthly. Burdett's "Hospital Annual," published at 428, Strand. A letter in "Everybody's Opinion" gives useful hints for nurses going to America.

(71) *Technical Terms (Nurse May).*—"The Nurses' Dictionary" by Honnor Morten, 428, Strand, price 2s.

(72) *Abroad (E. A. H.).*—Miss Bryant, at San Remo, and others, are mentioned in Burdett's "Hospital Annual."

(73) *One Year's Training (J. S. R.).*—You had better try a country hospital. We do not think any London hospital would take you in that way. Read "How to Become a Nurse." Perhaps you could get helpful information from the Secretary, Q.V.J.I., St. Catherine's Hospital, Regent's Park.

(74) *Would-be Probationer (Kate).*—It might be of general use to you, but would have no weight in that particular instance. You had better study "How to Become a Nurse," by Honnor Morten, price 2s. 6d., at Scientific Press, 428, Strand.

### The International Nursing Congress.

### MRS. BEDFORD FENWICK'S POSITION.

In connection with the information regarding the International Nursing Congress, which appeared in these columns on December 3rd, 1892, January 28th, and February 11th and 25th, 1893, we have received the following letter:—

3, Old Serjeants' Inn, Chancery Lane,
London, March 1st, 1893.

Sir,—In 1887, Mrs. Bedford Fenwick had occasion to instruct me to give you notice, that if you published any libel concerning her in The Hospital newspaper immediate legal proceedings would be taken against you.

You have since then prudently confined yourself to abuse and misrepresentation of every scheme with which Mrs. Fenwick has been identified.

In consequence of an article in The Hospital of February 25th, headed "Mrs. Bedford Fenwick's Position," a matter which is clearly no concern of yours, and which is discussed in a manner manifestly malicious, I am instructed now to give you formal notice that if any libel on Mrs. Bedford Fenwick is published in The Hospital newspaper, criminal proceedings will be immediately commenced against you and your co-editors and the printers of that paper without any further warning.—I am, Sir, your obedient servant, F. S. Randolph.

We have simply stated facts in regard to Mrs. Bedford Fenwick, which are supported by documentary evidence. We utterly disclaim all malice of every kind, and there is an entire absence of abuse and misrepresentation in the articles in question. The subject very much concerns the conductors of The Hospital, which is recognized as the most widely circulated and independent journal dealing with hospital and nursing matters.

Mr. Randolph used to threaten us by his letters at frequent intervals, but since his note of May 20th, 1889 (see The Hospital, May 25th, 1889), in which he said that "he was instructed to take immediate proceedings for libel whenever it may be necessary to do so," and a further one (see The Hospital for June 7th, 1890), we have been freed from similar communications. We referred Mr. Randolph at that time to our solicitors, Messrs. Slaughter and May, Austin Friars, E.C., who are prepared at any time, as they have always been, to accept service of any process which Mr. Randolph may at last summon up courage to advise his clients to bring against this journal or anyone connected with it. The press in England is not to be silenced by such threats as those contained in Mr. Randolph's letter. Further comment is superfluous.

### City Asylum, Birmingham.

The following nurses and attendants successfully passed the St. John Ambulance examination:—

First Aid.—Nurses: Holden, J. Berks, Vaughan, Coles, L. Wood, A. Jones, Houlston, Cooling, E. Moore, E. Edmunds, M. Edmunds, Saunders, Buncle, Simson, Warren, Keen, Freer, G. Jones, Darby, Gardener, and Little. Attendants: Bamford, Yarnal, Jones, Hill, Brindley, Withers, Drew, Underhill, Edwards, Baker, and Walthew.

### Minor Appointments.

London Hospital.—Miss Edith Helen Owen, who was trained at this hospital, has been appointed Sister of Cotton Ward.

West Ham Hospital.—Miss Ethel M. Willmott has been appointed Night Sister at the West Ham Hospital, Stratford, London, E. Miss Willmott trained at the Boston Hospital, Lincolnshire, under the late Miss K. M. Heanly, and has for some months had charge of wards at the Devon and Exeter Hospital.

### For Reading to the Sick.

### THE YOKE.

Lying on a sick bed, weak and feeble, after a sharp attack of illness, the future looms sadly before us. The burdens of life seem heavier and heavier as we think about them, and the question arises, How shall we carry them now we are less fitted for the effort?

"Take My yoke upon you and lean on Me," says our Lord's gentle voice; but the impatient heart replies, "We have quite enough troubles already, without adding another to our stock; why should we have grievous devotions, painful observances on our already wrung shoulders?" But let us pause and consider what we are saying. First, we will make up our minds that every one born into the world has burdens to carry; there is no shirking them till death bids us lay them down, but it is the part of Christianity to make them tolerable. And when we set ourselves up against the yoke, it is because we do not realise what it is, for we either imagine it to be the iron worn by the Roman soldier or the bondage of the Israelites in Egypt, or the subjection which "is good for a man to wear in his youth," whereas our Lord meant the simple "harness" or "ox-collar" of the Eastern peasant. It is the literal wooden yoke which He, as a modern writer says, "probably often made with His own hands in the carpenter's shop." "He knew that a plough without it would be torture; worked by means of a yoke it is light." He knew, too, the difference between a smooth yoke and rough one, a bad fit and a good fit—the difference it made to the patient animal that had to wear it. The rough yoke galled, and the burden was heavy; the smooth yoke caused no pain, and the load was lightly drawn. The badly-fitted harness was a misery, the well-fitted collar was "easy."

And yet with all this help close at hand we go on blindly, trying to do our work in our own way, taking no notice of Christ's offer of help It all lies in how we carry our burdens, and the whole world has ever been and ever will be taken up with trying to solve the problem, the only answer to which is given us by Christ Himself. He says, "Carry it as I do. Take life as I take it. Look at it from My point of view. Interpret it upon My principles. Take My yoke and learn of Me, and you will find it easy. For My yoke is easy, works smoothly, sits right upon the shoulders, and therefore My burden is light."

Oh! happy heart, that goes lightly on its way, fitted with a collar made by Christ's own Hands. He will show us where the old misfitting yoke hurts, and taking the pressure from the sore, will pour in the Balm of Gilead as He says, "Learn of Me, for I am meek and lowly of heart, and ye shall find rest unto your souls."

### Our Patient.

We nursed him through long weary hours of pain,
And fondly hoped to win him back again
To life, and strength, and all that he had been,
For nobler form, or comelier face, methinks were seldom seen.

But Death, more strong, perhaps more truly wise than we,
Gently but firmly drew him from our clasp, and he
Has gone to be with God, with Him to dwell,
And so we meekly bow our heads and say that "All is well!"

Nurse Beatrice Furnival.

### Where to Go.

"The Prevention of Epidemic Diseases, with especial reference to Cholera," is the title of a lecture by L. S. Trevor, Esq., M.R.C.S., L.R.C.P., announced for Friday evening, March 24th, at eight o'clock. It is to be given at the Trained Nurses' Club, 12, Buckingham Street, Strand. A few tickets for non-members can be obtained at sixpence each.

### The Muses' Looking Glass.

"THE DEATH OF ŒNONE."

This little volume has stirred in the nation a mingled passion of welcome and regret which finds its only parallel in the reception of "Asolando" nearly three years ago. It can only be handled just now with reverence. Later on, incorporated in the complete works, these few poems may lose something of the glamour as of last words which hangs over them and fall under the canon of criticism. To-day no one is concerned with the more or the less of beauty and wisdom. The leaves will be turned eagerly, yet the end reached all too soon, for not again will one of the familiar green volumes tempt the eye to glance between the uncut leaves in delighted anticipation of some new suggestion of beauty, with the certainty of being drawn for a few moments "above the smoke and stir of this dim spot." Old friends are best, all will uphold, yet no sober certainty of tried comradeship yields a pleasure so keen as the first unexpected flash of sympathy in dawning friendship. So no lingering over familiar verses, weighted with association, can rouse a glow of interest, a sense of talking face to face, such as the Laureate's latest words have been wont to inspire. And now the latest words are the last words.

"The Death of Œnone," which gives its name to the volume, is full of suggestions of the poet's earlier manner. The legend is derived from an epic poem by Quintus Smyrnæus, a poet of the fourth century, who saw the tale of Troy varied in some points from Homer and Virgil. It is a poem of desolation and widowhood. The vines which garlanded the abode of Œnone on Mount Ida in the happy days of her early marriage with Paris are withered—

And thro' the sunless winter-morning mist
In silence wept upon the flowerless earth.

She sits deserted, dwelling on the past—on the shepherd Paris, as she remembered him before the beauty of Helen had eclipsed her in his sight. So pondering, Paris stands before her, wounded to the death, imploring the succour she only can afford.

"Œnone by thy love which once was mine
Help, heal me. I am poisoned to the heart."
"And I to mine," she said; "Adulterer,
Go back to thine adulteress and die."

And still she sits on, crouching in her dark ravine while Paris passes from her and dies in the valley beneath. And still through the long day she stays motionless, "like a creature frozen to the heart," while the shepherds find their former comrade and build his funeral pyre, and lay him on it reverently. Then she sleeps and hears in her dream his voice calling to her. Then passing down the valley she sees the burning pyre and learns the truth—

and all at once
The morning light of happy marriage broke
Thro' all the clouded years of widowhood
And muffling up her comely head, and crying
"Husband!" she leapt upon the funeral pile,
And mixt herself with *him* and past in fire.

"St. Telemachus" gives the story of the Eastern hermit who brooded in the solitude of his prayers and fasts on the tales of the Roman gladiators which had reached him, until there came a vision of shadowy combatants and a voice calling—

"Wake
Thou, deedless dreamer, lazying out a life
Of self-suppression, not of self-less love."
And at his ear he heard a whisper, "Rome."
And in his heart he cried, "The Call of God."

Passing on foot to Rome he reached at length the Colosseum see

The gladiators moving towards their fight,
And eighty thousand Christian faces watch
Man murder man.

"The Death of Œnone." By Lord Tennyson. (Macmillan and Co.)

Roused at the sight to supernatural strength, he dashed through the spectators, passed the barrier,

And flung himself between
The gladiatorial swords, and called, "Forbear,
In the great name of Him who died for men,
Christ Jesus."

Telemachus was stoned to death by the enraged crowd; but the deed coming to the knowledge of Honorius, the combats of the circus were stopped for ever. And thus

His dream became a deed that woke the world.

Two tales of woman's heroism—one from the Sandwich Islands a hundred years ago, and one of our own land and day—are strangely contrasted.

To Kapiolani belongs the glory of triumph in her Master's name over superstition and priestcraft. For daring the double curse hurled on whoever should touch the berries or ascend the volcano sacred to the terrible goddess Peeli, Kapiolani

Baffled her priesthood,
Broke the taboo,
Dipt to the crater.

Called on the power adored by the Christian, and, crying "I dare her, let Peeli avenge herself!"
Into the flame billow dashed the berries, and drove the demon from Hawai-ee.

Was the heroism less which led the girl widow to seek out and save the woman loved and betrayed by her dead husband? The story is told by the ruined woman—

She had found my letter upon him, my wail of reproach and scorn;
I had cursed the woman he married, and him, and the day I was born.

And learning the truth, the young widow sought her out in her hour of shame, and nursed her back to life until, saved in body and soul, the outcast learns the true name of—

The tenderest Christ-like creature that ever stept on the ground;
She died of a fever, caught, when a nurse, in a hospital ward.
She is high in the Heaven of Heavens, she is face to face with her Lord,
And He sees not her like anywhere in this pitiless world of ours!
I have told you my tale. Get you gone, I am dressing her grave with flowers.

Many will linger over the shorter poems which close the volume, in various cadence of the voice, which has ever sung "the larger hope."

And let not reason fail me, nor the sod
Draw from my death Thy living flower and grass,
Before I learn that Love, which is, and was
My father, and my brother, and my God!

The "Silent Voices" continues the line of thought which would leave those things which are behind, calling—

Forward to the starry track,
Glimmering up the heights beyond me,
On, and always on!

More sure is the note of encouragement in "God and the Universe," which follows—

Fear not thou the hidden purpose of the Power which alone is great,
Nor the myriad world, His shadow, nor the silent Opener of the Gate.

And full of new and beautiful significance the lines on the death of the Duke of Clarence fitly close the long series, stretching over more than half a century, from the hand of him who began his life work as here he ends it, with bidding men

Mourn in hope.

### Wants and Workers.

The paragraph which appeared for two weeks in "Nursing News" headed "Wants and Workers," obtained a variety of answers, and Miss Coleman hopes these will result in her obtaining for her little hospital the help so urgently needed.

A home wanted for a gentleman in the country at once. He could make small payment. Suffers from occasional fits. Address Sister, care of Editor, 428, Strand, W.C.

### The Book World for Women and Nurses.

[We invite Correspondence, Criticism, Enquiries, and Notes on Books likely to interest Women and Nurses. Address, Editor, THE HOSPITAL (Nurses' Book World), 428, Strand, W.C.]

### TO OUR READERS.

With the view of meeting the wishes of a large circle of readers, we have determined to devote a special section of the "Nursing Mirror Supplement" to books and literary matters especially calculated to interest women and nurses. We are aware that, to very many, a careful classification of the book advertisements proves most helpful, and we shall endeavour to so arrange as to enable every reader of THE HOSPITAL to have information concerning every new book found of value. We shall be glad to give insertion to inquiries concerning rare or out-of-the-way books which may have a special interest of their own. We shall also welcome the co-operation of our readers to the extent of directing our attention to any British, Foreign, Colonial, or American books calculated to prove of service to women and nurses. Publishers will please direct all books intended for review to the Editor, 428, Strand, W.C.

### LEPROSY AND VACCINATION.*

Mr. William Tebb has written a controversial book. Now controversy is the soul of progress if conducted in a fairly judicial spirit. We say fairly judicial, because we can hardly expect the enthusiastic advocate to be as severely impartial as the judge. Mr. Tebb executes a strategic movement: his aim seems to be to attack vaccination on its flank, or even to take it in the rear. For our part we have no objection to this whatever. If vaccination be worth defending, it is as necessary to defend it on its flank or in its rear, as to resist an attack in front.

Mr. Tebb takes up several well-defined positions. For example, he affirms that there has been a large increase in the number of lepers in certain British dependencies and other countries during the last half-century, that this increase has been concurrent with the enforcement of general vaccination, that the leprosy is due to the vaccination, and that the practice of compulsory vaccination is, therefore, wicked and abominable, and ought to be exterminated root and branch.

Not so fast Mr. Tebb! The judge and the jury who are anxious to arrive at the justice of the case always take a good look at the witness. Mr. Tebb is the witness in the present case. He is a popular witness, not a scientific specialist. That is a fact to be taken into consideration, and more especially when the subject under discussion is of a purely scientific nature. Moreover, in the expressive American phrase, the witness has "an axe to grind." He has ends to serve which are personal to himself, in that he is the apostle and high priest of anti-vaccination. Therefore must his testimony be all the more severely scrutinised. Bearing in mind these modifying circumstances, we may proceed to the consideration of the subject matter of Mr. Tebb's contentions.

There has been a wide-spread increase of leprosy in certain parts of the British dominions, more especially in India and the West Indies, affirms the writer. On this point Mr. Tebb

* "Leprosy and Vaccination." By WILLIAM TEBB. (London: Swan Sonnenschein and Co.)

### THE BOOK WORLD FOR WOMEN AND NURSES—*continued.*

has gathered many facts and more opinions, and he has also made personal investigations on the spot. These are sound methods, and he is to be congratulated upon having put them into practice. The conclusion he has arrived at, namely, that there has been an increase in the number of cases of leprosy in the countries under discussion, seems to be quite sound, and we have no desire to call it in question. By way, however, of taking a juster measure of the extent of the increase than Mr. Tebb appears to have taken, we may point out that compulsory inspection and segregation were introduced at about the same period as vaccination, and that the probability is that much of the supposed increase in the number of lepers is not an increase in the actual number of cases, but in the number of cases discovered and brought under observation and control. So much for Mr. Tebb's first two points.

Leprosy has been spread in many of the countries under discussion by vaccination, and that, it is said, to a most distressing extent. Now it is obvious that this is a question of the utmost possible gravity. It is the duty of medical men, above all others, to see the facts in their true proportion and in their true light. In a matter of such moment the medical profession cannot afford to have any "axe to grind." Our character for scientific competency on the one hand, and for common humanity on the other, is at stake. We approach the question, therefore, with a due sense of responsibility.

Has leprosy been spread by vaccination; and if so, to what extent? Before answering these questions it is necessary to ask another, viz.: Can leprosy or any such disease be spread by vaccination? Twenty or thirty years ago scientific opinions might have differed on this point. But modern bacteriology teaches us very clearly that leprosy can be contracted by means of vaccination; nay more, that in all probability inoculation is the chief, if not the only, method by which it is spread. Mr. Tebb's book offers evidence of a convincing character to the effect that leprosy has been contracted as the result of vaccination, and that to a considerable extent in several countries.

From these premisses Mr. Tebb jumps to the conclusion that compulsory vaccination is bad and ought to be suppressed. The conclusion to the untrained reader may seem very obvious; so obvious, indeed, that it ought at once to excite suspicion in the scientific mind. Granted that leprosy has been spread by vaccination, does it necessarily follow that vaccination itself has been at fault? Does vaccination always impart leprosy? The idea is ridiculous! How many of the millions who have been vaccinated in this country have displayed any symptoms of leprosy? The truth seems to be that the number of vaccination lepers is very much smaller than Mr. Tebb would have us believe, and that the majority of cases of leprosy due to vaccination owe their origin to the want of reasonable care on the part of the vaccinators.

The proper conclusions to be derived from Mr. Tebb's facts and gathered opinions are that leprosy may be communicated by means of vaccination; and that, therefore, the vaccinator should exercise the utmost possible care in the selection of the children from whom he derives his lymph. It might be left to the discretion of medical men in leprosy districts to abstain from vaccinating altogether rather than to run the risk of vaccinating with leprous lymph.

Mr. Tebb's book does not touch the general question, scientifically at any rate, of the value of vaccination as a prophylactic against small-pox; nor are its facts and opinions of any weight upon the subject of compulsion in this country, where leprosy is hardly known to exist. As for the West Indies and certain other of our dependencies where small-pox has been one of the most deadly of scourges for many decades, it seems eminently desirable that vaccination shall continue to be practised, and that it shall be compulsory. But this condition must always be insisted upon, that it shall be performed with perfectly pure lymph. If that be done we shall hear no more of leprosy spread by vaccination.

